# The strategies of NLRP3 inflammasome to combat *Toxoplasma gondii*

**DOI:** 10.3389/fimmu.2022.1002387

**Published:** 2022-10-19

**Authors:** Chanjin Yoon, Yu Seong Ham, Woo Jin Gil, Chul-Su Yang

**Affiliations:** ^1^ Department of Molecular and Life Science, Hanyang University, Ansan, South Korea; ^2^ Center for Bionano Intelligence Education and Research, Ansan, South Korea

**Keywords:** *Toxoplasma gondii*, NLRP3, inflammasome, rhoptry, dense granule

## Abstract

Infection with the protozoan parasite *Toxoplasma gondii* (*T. gondii*) results in the activation of nucleotide-binding domain leucine-rich repeat containing receptors (NLRs), which in turn leads to inflammasome assembly and the subsequent activation of caspase-1, secretion of proinflammatory cytokines, and pyroptotic cell death. Several recent studies have addressed the role of the NLRP3 inflammasome in *T. gondii* infection without reaching a consensus on its roles. Moreover, the mechanisms of NLRP3 inflammasome activation in different cell types remain unknown. Here we review current research on the activation and specific role of the NLRP3 inflammasome in *T. gondii* infection.

## Overview of NLRP3 inflammasome

The immune system has two subsystems, the innate and adaptive immune systems, that work together to protect the body from pathogens, such as bacteria, viruses, fungi, and parasites (1, 2). The innate immune system is the initial line of defense, and its quick response to pathogens involves the recognition of pathogen-associated molecular patterns (PAMPs) or danger-associated molecular patterns (DAMPs) by pattern recognition receptors (PRRs) (3). Based upon their subcellular localization, PRRs are classified into (i) Toll-like receptors (TLRs) and C-type lectin receptors (CLRs) located in the plasma membrane and (ii) cytoplasmic PRRs. TLRs and CLRs recognize extracellular PAMPs and DAMPs. Cytoplasmic PRRs, on the other hand, involve retinoic acid-inducible gene I (RIG-I)-like receptors (RLRs), AIM2-like receptors (ALRs), nucleotide-binding and oligomerization domain (NOD)-like receptors (NLRs), and the cytosolic sensor cyclic GMP-AMP (cGAMP) synthase (cGAS) (4, 5). The signaling cascades triggered by PRRs play crucial roles in the immune response, including antigen presentation, cell death, and cytokine secretion (6–8).

The inflammasomes are large multi-protein complexes whose assembly is initiated by the different cytoplasmic PRRs, such as NLRs and ALRs (9). The proteins in the NLR family are derived from 22 human genes and share common structure motifs consisting of C-terminal leucine-rich repeat domains and central nucleotide-binding domains (NBDs) (10). (NBD is a component of the larger NACHT domain). According to their variable N-terminal domain, the NLR family has been classified into a variety of subfamilies, including NLRCs, whose N-terminal have one or more caspase-recruitment domains (CARDs), and NLRPs that have pyrin domains (PYD) instead of CARD. NLRC and NLRP are the two most characterized NLR subfamilies (11).

The NLRP3 is one of the most well-studied and characterized protein in the NLR family. The activation of the NLRP3 inflammasome requires signaling in two steps, with a priming signal in the first and an activation signal in the second. The first, priming, step is initiated through the recognition of the various PAMPs or DAMPs by PRRs, such as TLRs or nucleotide-binding oligomerization domain-containing protein 2 (NOD2) or interleukin 1 receptor, type I (IL1R1), that lead to nuclear factor-κB (NF-κB) activation (12). The NF-κB, once activated, boosts the transcription and expression of inflammatory cytokines, including pro-IL-1β and pro-IL-18 (13). The second, activation, step is triggered by not only bacteria, viruses, and fungi but also sterile inflammation mediated by DAMPs or various other stimuli, including ionic flux, mitochondrial dysfunction, production of reactive oxygen species (ROS), and lysosomal damage (14–16).

NLRP3 activation leads to the assembling of an oligomeric protein complex that includes an adaptor protein called apoptosis-associated speck-like protein containing a caspase-recruitment domain (ASC) and an effector protein, pro-caspase-1 (17). Upon Inflammasome activation, the PRRs, such as NLRs and ALRs, are recruited to bind with the ASC through PYD (18). Then PRR-ACS complexes, interacting through their CARDs, recruit pro-caspase-1 (19), converting them into active caspase-1 by proteolytic cleavage (20). In turn, the activated caspase-1 promotes the cleavage of precursor cytokines, such as pro-IL-1β and pro-IL-18, to generate active cytokines (IL-1β and IL-18), and then facilitates their secretion (6, 9). Furthermore, the activated caspase-1 triggers pyroptosis, an inflammatory cell death, through the cleavage of the protein gasdermin D (GSDMD) (21).

Several studies have reported that the NLRP3 inflammasome responds to bacterial pathogens (22–24). More recent studies have reported that the NLRP3 inflammasome also plays a crucial role in the host’s response to protozoan parasite infection (25–27). This review focuses on the current understanding of the immune response mechanisms that involve the NLRP3 inflammasome in *T. gondii* infection.

### Toxoplasma gondii

*Toxoplasma gondii* is a protozoan parasite that is estimated to infect at least one-third of the worldwide human population (28). *T. gondii* infection is typically asymptomatic in immunocompetent individuals. However, *T. gondii* infection may contribute to focal central nervous system disease and other severe diseases in immunodeficiency syndrome (AIDS) patients and poses a risk of congenital infection of a newborn baby by the mother who got infected during the pregnancy period (29). Because *T. gondii* can infect nearly all nucleated cell types in most species of warm-blooded animals, the range of *T. gondii* hosts is extremely broad.

## The function of the NLRP3 inflammasome in various cell types infected with *T. gondii*

To resist *T. gondii* infection, components of the innate and adaptive immune system are recruited in various cell types. These include inflammatory monocyte, dendritic cells (DCs), neutrophils, macrophages, NK cells, and Th1 cells (30). TLRs are responsible for the initial detection of *T. gondii*. Resistance to *T. gondii* infection is mediated by the TLR-associated adaptor protein MyD88 and the induction of IL-12, interferon-γ (IFN-γ), and the synthesis of nitric oxide (NO) (31). TLRs induce the activation of MyD88 which leads to a downstream signaling cascade recruiting host resistance. Sher et al. reported that IL-12 and IFN-γ responses were reduced but not completely abolished and parasite infection was highly susceptible in MyD88-deficient mice (31). These results indicate that, in addition to the crucial role played by MyD88, there were other mechanisms involved in the detection of *T. gondii* that remain to be identified.

Recently, Grigg et al. showed that, compared with C57BL/6J mice, NLRP3^−/−^ mice harbored greater parasite burdens and their IL-18 response was significantly reduced (32). In addition, Robson et al. reported that the P2X7 receptor (P2X7R) activated by extracellular ATP inhibited *T. gondii* growth through the NLRP3 inflammasome and produced reactive oxygen species (ROS) and IL-1β in murine macrophage (33).

Several studies reported that the sensitivities for activation of NLRP3 inflammasome were different in the human monocyte/macrophage and mouse macrophage. Chin and Kostura reported that, in humans, treatment with low-dose lipopolysaccharide (LPS) induced pro-IL-1β transcription, but treatment with a higher dose of LPS lead to the secretion of activated IL-1β (34). In contrast, both LPS priming and ATP induction are typically required for the NLRP3 inflammasome activation in mouse bone marrow-derived macrophages (BMDMs) (35). Additionally, Meng et al. showed that LPS treatment activated the NLRP3 inflammasome in the human monocyte cell line THP-1 but not in mouse BMDMs (36). Furthermore, despite the TLRs playing a crucial role in the detection of *T. gondii*, TLR11 is a pseudogene and TLR12 is not expressed in humans. Thus, NLRP3 inflammasome activation must involve different mechanisms in human and murine cells. Furthermore, in humans, *T. gondii*-induced NLRP3 inflammasome activation varies depending on the cell type.

Previous studies reported that *T. gondii* infection was implicated in the activation of NLRP3 inflammasomes and in stimulating IL-1β secretion in human monocytes. To directly address whether the regulation of IL-1β and IL-18 were responses to *T. gondii* infection in human monocytes, Lodoen et al. infected THP-1 and U937 cells, which are commonly used as models for human monocytes, with *T. gondii* and observed *T. gondii*-induced IL-1β response in both cell lines (37, 38). Primary human monocytes showed similar responses to those observed in the human monocytic cell lines (39). Specifically, *T. gondii* infection increases the rapid induction of NLRP3 transcription and IL-1β production. Conversely, treatment with MCC950, a selective small-molecule inhibitor of NLRP3, reduced IL-1β production compared with vehicle control (38). This study further suggested that potassium efflux was implicated in producing IL-1β since the production of IL-1β was significantly diminished in primary human monocytes after high potassium release (38). Another study reported that the increased IL-1β production in *T. gondii*-infected THP-1 cells depended on syk phosphorylation (40). The same study also showed that rapidly increased syk phosphorylation in human primary monocytes infected with *T. gondii* lead to a downstream cascade in the PKCδ-CARD9/MALT-1 pathway, subsequently leading to the activation of NF-κB, which in turn produces pro-IL-1β and NLRP3 (40). Taken together, these lines of evidence support the notion that IL-1β secretion in human monocytes induced by *T. gondii* infection requires the NLRP3 inflammasome through potassium efflux or the PKCδ-CARD9/MALT-1 signaling pathway.

Interestingly, the responses to the NLRP3 inflammasome in human macrophages were different from those in human monocyte during *T. gondii* infection. To investigate the role of the various inflammasome components in PMA-differentiated THP-1 macrophages infected with *T. gondii*, Quan et al. measured the expression of inflammasome-associated genes and the secretion of inflammatory cytokine IL-1β. The investigators reported that *T. gondii* infection significantly increased the expression of IL-1β in PMA-differentiated THP-1 macrophages. Furthermore, mRNA expressions levels for inflammasome sensors, including NLRP1, NLRP3, NLRC4, NLRP6, NLRP8, NLRP13, AIM2, NAID, ASC, and caspase-1, were significantly elevated over the levels in mock-infected THP-1 macrophages (41). In contrast, primary human macrophages infected with *T. gondii* did not secrete IL-1β, and NLRP3 was downregulated in these cells (40). Although these studies were unable to fully identify the difference in the mechanisms of *T. gondii* infection induced NLRP3 inflammasome activation between human macrophage cell lines and primary human macrophages, there are some clues for an explanation. Huang et al. showed that levels of NLRP3, caspase-1, and IL-1β were significantly elevated in PMA-differentiated THP-1 macrophages (42). In contrast, Karabina et al. reported that NLRP3, NLRP6, and NOD2, whose normal levels are already low in peripheral blood mononuclear cells, were further decreased during the differentiation of primary human monocytes to macrophages (43). This raises two possible explanations: either (i) *T. gondii* was not sufficient to trigger the expression of inflammasome components in primary human macrophages because these cells require a stronger signal to respond to *T. gondii* infection, or (ii) the primed state of human macrophage cell lines and primary human macrophages may be different (38).

*T. gondii* infection in FHs 74 Int cells, a human fetal small intestinal epithelial cell line, significantly increased NLRP3 activation, subsequently leading to IL-1β production. Furthermore, *T. gondii* infection of these cells activated P2X7R, whereas silencing P2X7R significantly reduced *T. gondii*-induced IL-1β secretion as well as *T. gondii* proliferation (44). In addition, *T. gondii*-induced NLRP3 inflammasome activation in FHs 74 Int cells involved the phosphorylation of both p38 MAPK and JNK1/2, even though the p38 MAPK pathway has a more crucial role than the JNK1/2 pathway (45).

Human neutrophils, which T. gondii manipulates to evade innate immunity, present a special case. Lodoen et al. showed that *T. gondii* infection in the human neutrophils inhibited LPS-induced IL-1β and NLRP3 transcription and decreased the expression of pro-IL-1β, mature IL-1β, and the NLRP3 inflammasome by inhibiting both NF-κB signaling and the activation of the NLRP3 inflammasome (46) (**Table 1**). However, as other mechanisms and pathways involving NLRP3 remain unclear, further experiments are required to fully understand the role of NLRP3 in *T. gondii* infection.

**Table 1:** Mechanisms of NLRP3 inflammasome regulation in various *T. gondii*-infected cell types.

Species	Cell type	NLRP3 inflammasome	Comments	Ref.
Mouse	Primary macrophage	Activated	Activation of NLRP3 by P2X7 receptor	(33)
Human	Monocyte cell line		Induction of IL-1β mRNA expression and secretion	(38)
			syk phosphorylation-dependent IL-1β production	(38)
	Primary monocyte	Activated	NLRP3 and potassium efflux-dependent IL-1β secretion	(40)
		Activated	NLRP3 activation by Syk-CARD9/MALT-1-NF-κB signaling pathway	(40)
	Macrophage cell line	Activated	Induction of NLRP3 mRNA expression and IL-1β secretion	(41)
	Primary macrophage	–	No change in IL-1β and NLRP3 expression	(38)
	Fetal small intestinal epithelial cell line	Activated	NLRP3-dependent IL-1β secretion by P2X7 receptor	(44)
		Activated	p38 MAPK and JNK1/2 pathways involved in NLRP3 activation	(45)
				
	Neutrophils	Inhibited	Inhibition of NLRP3 activation.	(46)

## The function of other NLR families in *T. gondii* infection

The study of NLR inflammasome activation related to the *T. gondii* infection was initiated by a report of the susceptibility alleles involved with human congenital toxoplasmosis. McLeod et al. reported that *T. gondii* progression was attenuated and proinflammatory cytokines, including IL-1β and IL-18, were not upregulated in the human monocyte with NLRP1 knockout engineered by RNA interference (47). These results implicated the NLRP1 inflammasome with a crucial role in limiting *T. gondii* replication and the production of pro-inflammatory cytokines.

Nuñez et al. reported that NOD2 deficiency results in a not fully functional immune response against *T. gondii* infection. Specifically, survival of *T. gondii* was impaired and IFN-γ secretion was reduced in Nod2^−/−^ mice. In addition, Nod2 promoted the generation of an effective Th1 response through T cell-intrinsic signaling against *T. gondii*. In contrast, Th cell differentiation was impaired in association with reduced IL-2 production and nuclear accumulation of the transcription factor c-Rel in Nod2^−/−^ mice (48).

AIM2 consists of a C-terminal HIN-200 domain, which interacts with cytosolic double-stranded DNA; it binds to a PYD domain of ASC and subsequently activates the AIM2 inflammasome (49). AIM2 detected GPT-promoted *T. gondii* and induced atypical apoptosis through ASC and Caspase-8 in PMA-differentiated THP-1 macrophages (50). Ahmadpour et al. reported that expression levels for NLRP12, caspase-3, caspase-1, IL-1β, IL-18, and ASC mRNA were significantly increased in mice injected with *T. gondii* live tachyzoites over levels in control mice. In addition, the intracellular ROS levels in tachyzoite-injected mice were significantly higher than in control mice. These results suggest that *T. gondii* infection may activate the NLRP12 inflammasome and increase the expression of proinflammatory cytokines such as IL-1β and IL-18 and activates the pyroptosis pathway (51).

In summary, despite the presence of several NLR families, only a few inflammasomes were identified. Thus, further investigations are required to understand the role of other NLR proteins, including NLRP1, NLRP3, NLRP12, AIM2, and NOD2, in *T. gondii* infection.

## Mechanism of pyroptosis and potential application in *T. gondii* infection

Pyroptosis is an inflammatory programmed-necrotic cell death that is induced by various stimuli such as bacteria, viruses, fungi, and protozoa (52). Pyroptosis pathways are typically classified into caspase-1-dependent canonical pyroptosis and human caspase-4/5 or mouse caspase-11-induced non-canonical pyroptosis (53, 54). Several studies have reported that *T. gondii* is involved with pyroptosis through a series of inflammatory reactions. Kimberly et al. reported that knockdown of Nlrp1 increases replication of parasites and protects against cell death in pyroptosis-sensitive macrophages from rats such as Lewis and spontaneously hypertensive (SHR) (55). Furthermore, Wang et al. reported that *T. gondii* upregulated the level of ROS induced by GRA43 resulting in peritoneal macrophages (PMs) pyroptosis in iNOS^−/−^-SD rats (56), which indicated that iNOS is considered to be a key factor that leads to pyroptosis in SD rat PMs. In contrast, pyroptosis does not occur in macrophages from T. gondii-susceptible strains of rats such as Brown Norway (BN), Sprague Dawley (SD), and Fischer (CDF), or *T. gondii*-infected mouse macrophages (32, 33, 55). In addition, human monocytes infected with *T. gondii* were independent of GSDMD cleavage and pyroptosis, despite increased IL-1β production *via* the NLRP3 inflammasome. Moreover, GSDMD knockout THP-1 cells secreted a not statistically significant amount of IL-1β production compared with wild-type THP-1 cells after *T. gondii* infection (40). Taken together, *T. gondii* might manipulate the host cell niche to maintain the integrity of host cells and immune escape, although the host cell produces a protective cytokine.

Recently, an elevating incidence rate of clinical diseased, including atherosclerosis and cancer, have been reported to be involved with pyroptosis and play a crucial role in pyroptosis (57). Pyroptosis reveals not only a protective effect against pathogens but also beneficial effects on tumor suppression. Wang et al. reported that less amount of pyroptosis cell death was sufficient to clear the entire tumor graft (58). Furthermore, several studies reported that granzymes from cytotoxic lymphocytes cleave GSDMB or GSDME to trigger pyroptosis and potently suppress tumor growth (59, 60). However, the relationship between pyroptosis and *T. gondii* remains unclear, further experiments are required to understand and clinical application of pyroptosis will be one of the successful non-surgical treatments.

## NLRP3 response to *T. gondii* effector proteins

**Figure 1:**
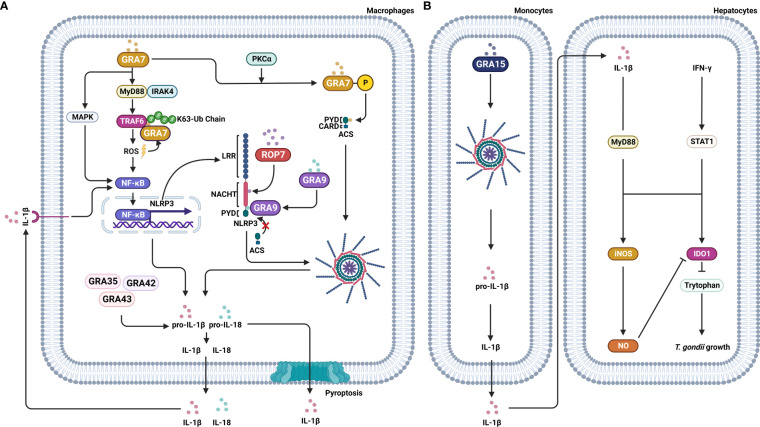
Effector proteins of *T. gondii* manipulate signaling pathways of the host cell. *T. gondii* secrets effector proteins, including ROP and GRA, into the host cell to influence the signaling pathways of the host. **(A)** ROP7 of *T. gondii* interacts with the NACHT domain of NLRP3 and assembles the NLRP3 inflammasome, leading to IL-1β secretion, which in turn induces an NF-κB/NLRP3-positive loop. GRA7 induces the expression of proinflammatory cytokine IL-1β through MyD88-dependent ROS generation and TRAF6 activation. GRA7, after PKCα-mediated phosphorylation, interacts with the PYD domain of ACS, inducing ASC oligomerization and inflammasome activation. GRA35, GRA42, and GRA43, induce pyroptosis and IL-1β secretion. In contrast, GRA9 binds with NLRP3, which results in blocking the interaction between ASC and NLRP3 and, in turn, the disrupting of the NLRP3 inflammasome assembly. **(B)** GRA15 induces IL-1β secretion through the NLRP3 inflammasome. Secreted IL-1β and IFN-γ induce iNOS expression and NO production, resulting in the reduction of IDO1 expression and contributing to *T. gondii* growth in hepatocytes.

*T. gondii* secretes numerous effector proteins from the rhoptry (ROP) and dense granule (GRA) organelles, which are required to maintain an equilibrium between host immune responses and the parasite immune evasion, resulting in the survival of both host and parasite. The ROPs are secreted by *T. gondii* and injected into the host cell during or immediately before the invasion to form the parasitophorous vacuole (PV) with ROP neck proteins (RON) (61). After the invasion and the establishment of the PV, *T. gondii* secretes GRA proteins that are involved in modifying the PV and creating an environment for intracellular survival and replication (62). Several recent reports implicated ROP and GRA secreted by *T. gondii* with the inflammasome complex. For example, Cheng et al. reported that *T. gondii*-infected PMA-differentiated THP-1 macrophages significantly up-regulate NF-κB and the secretion of IL-1β, which, depending on the presence of *T. gondii* effector protein ROP7, lead to the assembly of inflammasome. In addition, ROP7 was shown to interact with the NACHT domain of NLRP3 to induce inflammasome hyperactivation through the IL-1β/NF-κB/NLRP3-positive loop (63). GRA7 stimulation induced the expression of pro-inflammatory cytokine genes, including IL-1β in BMDM, in mice (64). Further, the *T. gondii* protein GRA7, after PKCα-mediated phosphorylation, interacted with the PYD domain of ACS, facilitating ASC oligomerization and inflammasome activation (65). Similarly, Yang et al. reported that GRA9, another *T. gondii* effector protein, interacted with NLRP3 to block the interaction between ASC and NLRP3, disrupting the assembly of the NLRP3 inflammasome. GRA9 also showed a potential to protect against sepsis by increasing anti-inflammatory and anti-bacterial effects through the polarization of M1 to M2 macrophages (66). Yet another *T. gondii* effector protein, GRA15, induced IL-1β production and secretion through the NLRP3 inflammasome. The IL-1β derived from THP-1 cells, together with IFN-γ, induce iNOS expression and NO production, resulting in the reduction of IDO1 expression and contributing to *T. gondii* growth in hepatocytes (67) (**Figure 1**). Three effector proteins, GRA35, GRA42, and GRA43, play an important role in *T. gondii* infection by inducing pyroptosis and IL-1β secretion in Lewis rat BMDMs (68). Although the involvement of these effector proteins with NLRP3 has not been studied, several studies reported that effector proteins of *T. gondii* induce an antitumor response. In particular, Shen et al. reported that GRA15 induced macrophage polarization into M1 that suppressed several hepatic carcinoma cell characteristics, including proliferation, invasion, metastasis, and reduced the expression of matrix metalloproteinases (MMP-9 and MMP-2). In addition, both spleen tumor tissues and tumor growth were decreased in GRA15-polarized macrophages-injected tumor-bearing C57BL/6 mice, and IL-6 expression was also decreased in tumor tissue (69). Another study reported that secretion of several ROPs and GRAs induced an antitumor immune response in C57BL/6 mice with ovarian tumors. Deletion of the genes for ROP5, ROP17, ROP18, ROP35 and ROP38, GRA2 or GRA12, and GRA24 markedly impaired the antitumor response against ovarian tumors in mice (70). Previous reports indicated that effector proteins resist tumor progression *via* enhancing the immune response of hosts. However, recent reports suggested *T. gondii*-infection regulated the expression of tumor-involved factors, which are able to enhance the anti-tumor ability of hosts. TP53 is a critical tumor-suppressor gene that is frequently mutated in most types of cancer (71). Lu et al. reported that the p53 signaling pathway was altered through up-regulated Gadd45 protein and down-regulated Fas protein after *T. gondii* infection (72). Further, the colorectal cancer pathway involved genes which are DCC, Smad2, Smad4, hMLH1, hMSH2, and hMSH3 protein expression were regulated after *T. gondii* infection. In particular, DCC, the colorectal cancer suppressor, is a prognostic marker in patients with stage II or stage III colorectal cancer (73). The DCC protein expression was elevated after *T. gondii* infection. The same study also reported that RASSF1, non-small cell lung cancer (NSCLC) tumor suppressor, expression was elevated after *T. gondii* infection, which indicated that *T. gondii* infection might be improved NSCLC. PTEN, BRCA1, BRCA2, PI3K, and CCND1 genes were involved with the breast cancer signaling pathway. *T. gondii* infection decreased CCND1 expression and increased BRCA2 expression, which suggests that *T. gondii* infection could suppress breast cancer growth (72).

In summary, although the effector proteins of *T. gondii* have various functions implicated in the host immune responses and the antitumor immune response, the associated mechanisms are unknown, and understanding them will require further studies.

## Conclusion

Inflammasomes are involved with the immune response of the host combating *T. gondii* infection. Despite the considerable number of studies investigating inflammasomes implicated with *T. gondii*, only a few inflammasomes have been identified so far, pointing to the need for further investigations, focusing on additional inflammasome proteins involved in the detection of *T. gondii* by the host cells and the molecular mechanisms of the immune response. In addition, the responses of inflammasomes to *T. gondii* infection have been shown to depend on the host cell types. The reason for this is not well understood. It is hypothesized that NLRP3 inflammasome activation might have different requirements in different cell types. For instance, although ATP induces P2X7R signaling that implicates inflammasomes, ATP amount released differs with cell types (74). Moreover, several proteins involved in inflammasomes, such as CARD8, POP1, and POP2, are differentially expressed in species dependent way (36). Thus, future investigations will need new approaches using different perspectives, such as species and cellular characteristics.

*T. gondii* secretes effector proteins to invade the host cell and several these are known to be involved with inflammasomes. However, the function and mechanisms of action of the effector proteins of *T. gondii* remain unknown. Recent studies using transcriptome sequencing analysis of *T. gondii*-infected mice reported that differentially expressed genes involved in the p53 signaling pathway, colorectal cancer pathway, non-small cell lung cancer signaling pathway, and breast cancer signaling pathway were upregulated or downregulated (72). Other studies reported that effector proteins of *T. gondii* suppressed tumor growth and induced antitumor immune responses (69, 70). Others reported a protective effect of *T. gondii* effector proteins against sepsis through increasing anti-inflammatory and bacterial effects (66). Taken together, these results are consistent with the notion that the effector proteins of *T. gondii* act like a double-edged sword: support invasion during *T. gondii* infection but, depending on the application, they may be beneficial in the treatment of diseases, too. Therefore, future investigations should focus on the understanding of the effector proteins derived from *T. gondii* and their application to inflammasome-related diseases and cancer as well as other diseases. An understanding of inflammasome regulation in *T. gondii* infection suggests novel strategies for host immune response mechanisms and may provide opportunities to develop new treatments for various diseases.

## Author contributions

CY, YH, WG, and C-SY designed, conceptualized, and wrote the manuscript. All authors contributed to the article and approved the submitted version.

## Funding

This work was supported by a National Research Foundation of Korea grant funded by the Korea government (MSIP) (grant no. 2019R1I1A2A01064237 and 2021R1A4A5032463), by a grant of the Korea Health Technology R&D Project through the Korea Health Industry Development Institute (KHIDI), funded by the Ministry of Health & Welfare, Republic of Korea (HI22C0884).

## Conflict of interest

The authors declare that the research was conducted in the absence of any commercial or financial relationships that could be construed as a potential conflict of interest.

## Publisher’s note

All claims expressed in this article are solely those of the authors and do not necessarily represent those of their affiliated organizations, or those of the publisher, the editors and the reviewers. Any product that may be evaluated in this article, or claim that may be made by its manufacturer, is not guaranteed or endorsed by the publisher.
